# Supplementary figures and images for: High-intensity interval training remodels the proteome and acetylome of human skeletal muscle
Source: eLife. 2022 May 31;11:e69802. doi: 10.7554/eLife.69802 (PMC9154743; doi:10.7554/eLife.69802)

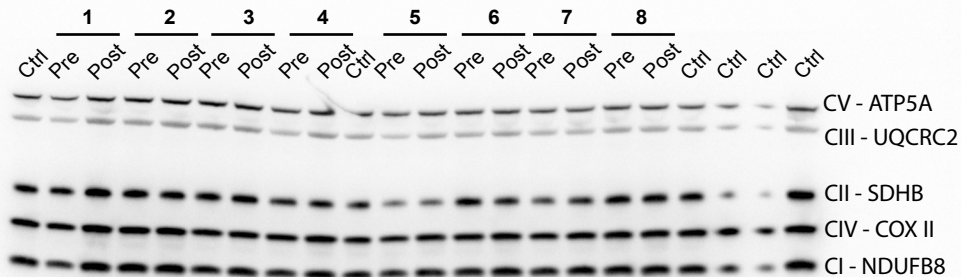

Supplement: Figure 2—figure supplement 1—source data 1. [file elife-69802-fig2-figsupp1-data1.pdf]

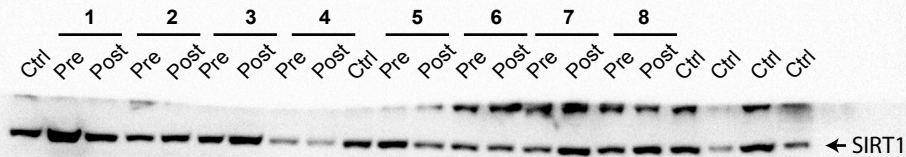

Supplement: Figure 5—source data 1. [file elife-69802-fig5-data1.pdf]

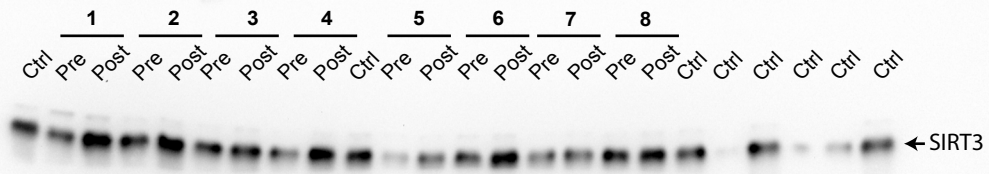

Supplement: Figure 5—source data 4. [file elife-69802-fig5-data4.pdf]

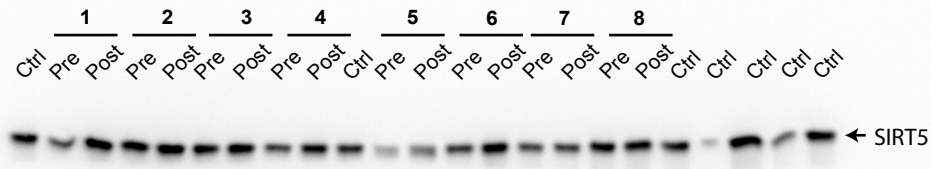

Supplement: Figure 5—source data 7. [file elife-69802-fig5-data7.pdf]

SIRT6 was measured on a membrane previously exposed for NAMPT

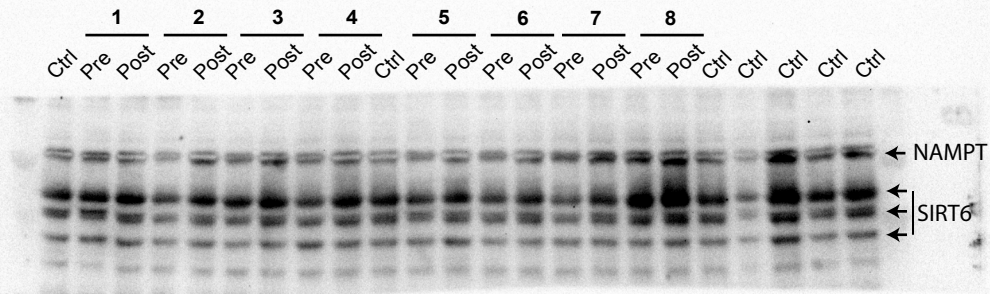

Supplement: Figure 5—source data 10. [file elife-69802-fig5-data10.pdf]

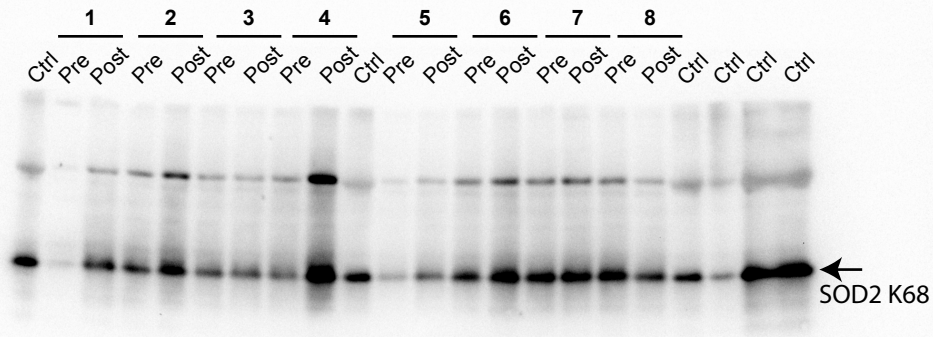

Supplement: Figure 5—figure supplement 1—source data 4. [file elife-69802-fig5-figsupp1-data4.pdf]

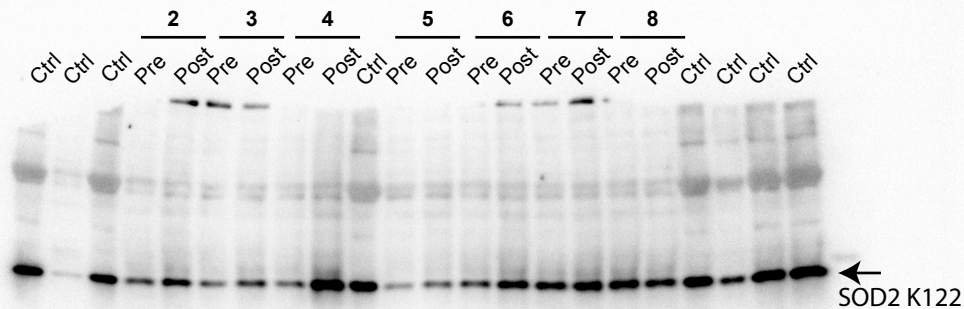

Supplement: Figure 5—figure supplement 1—source data 7. [file elife-69802-fig5-figsupp1-data7.pdf]

1 2 3 4 5 6 7 8  
Ctrl Pre Post Pre Post Pre Post Pre Post Ctrl Pre Post Pre Post Pre Post Ctrl Ctrl Ctrl Ctrl

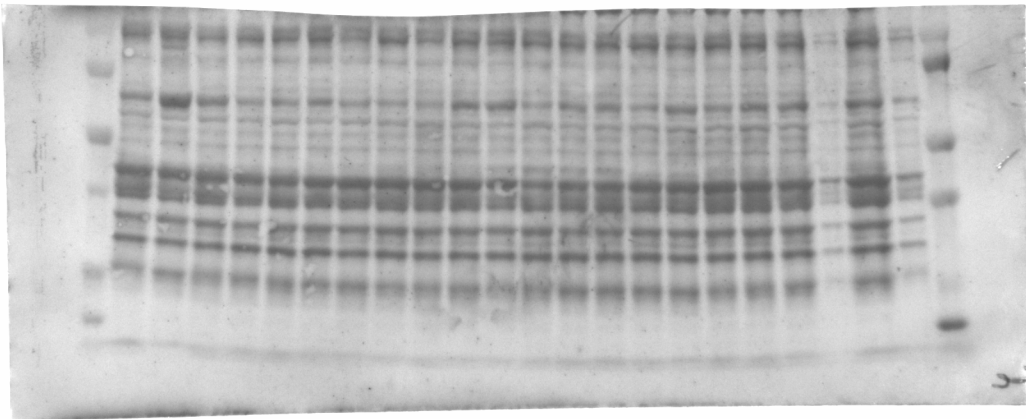

Supplement: Figure 5—figure supplement 2—source data 1. [file elife-69802-fig5-figsupp2-data1.pdf]

1 2 3 5 6 7 8  
Ctrl Pre Post Pre Post Pre Post Ctrl Ctrl Pre Post Pre Post Pre Post Ctrl Ctrl Ctrl Ctrl

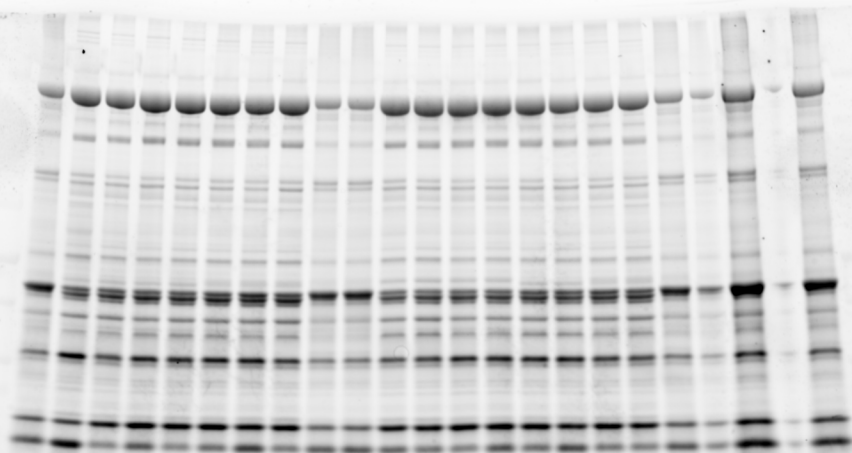

Supplement: Figure 5—figure supplement 2—source data 3. [file elife-69802-fig5-figsupp2-data3.pdf]

Samples not included  
in this study

EP300  
+ + + - - -

Samples not included  
in this study

EP300  
+ + + - - -

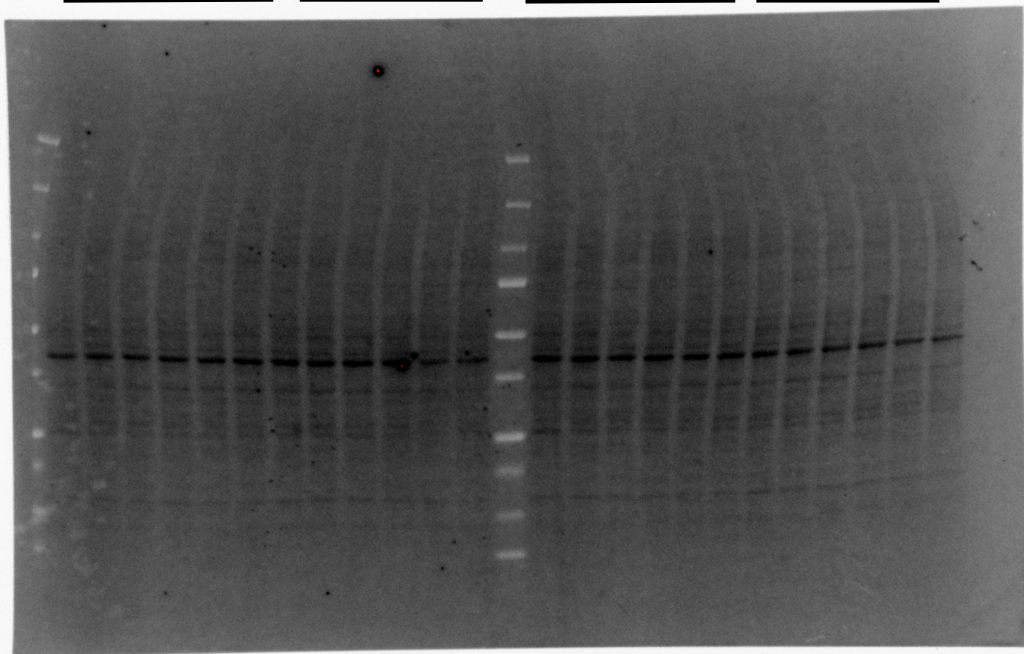

Lanes shown in Supp C

Supplement: Figure 5—figure supplement 2—source data 5. [file elife-69802-fig5-figsupp2-data5.pdf]

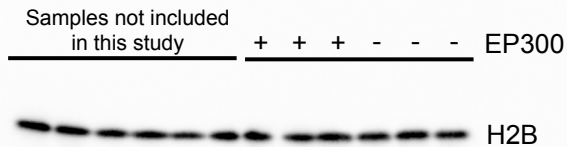

Supplement: Figure 7—source data 1. [file elife-69802-fig7-data1.pdf]

Samples not included  
in this study

---

+ + + - - -

---

EP300

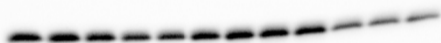

H2B K20

Supplement: Figure 7—source data 3. [file elife-69802-fig7-data3.pdf]

Samples not included  
in this study

+ + + - - -

EP300

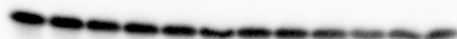

H3

Supplement: Figure 7—source data 6. [file elife-69802-fig7-data6.pdf]

Samples not included  
in this study

+ + + - - -

EP300

H3 K23

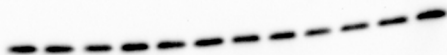

Supplement: Figure 7—source data 8. [file elife-69802-fig7-data8.pdf]

Replicate 1

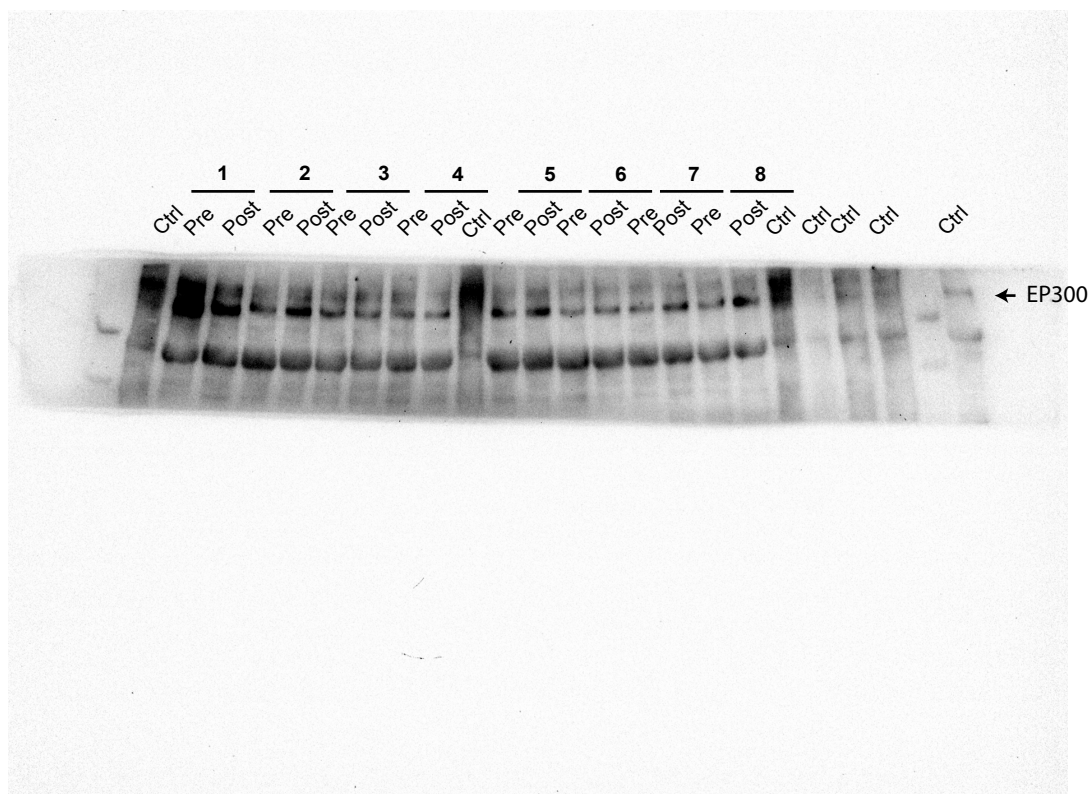

Replicate 2

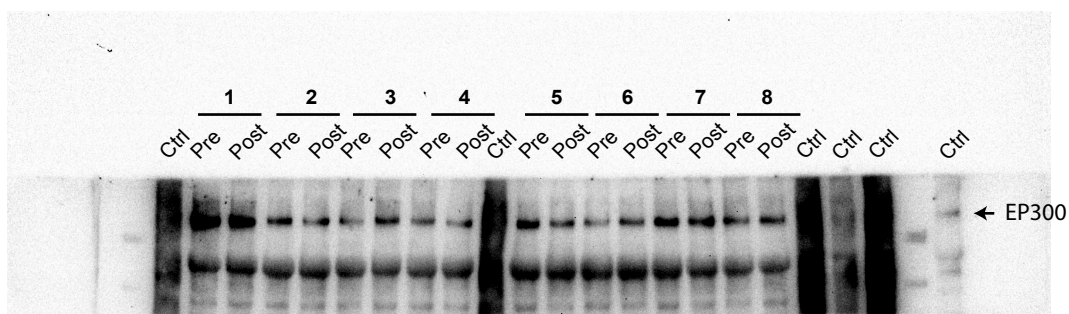

Replicate 3

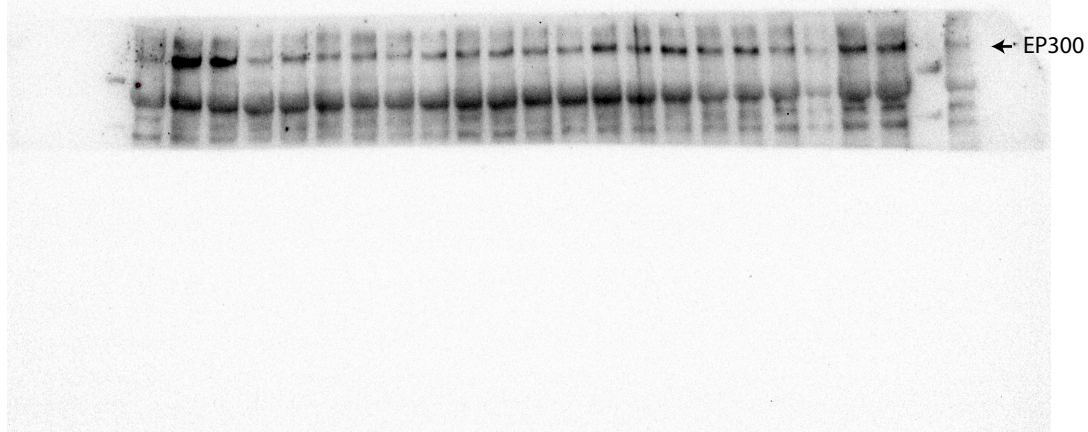

Supplement: Figure 7—source data 11. [file elife-69802-fig7-data11.pdf]

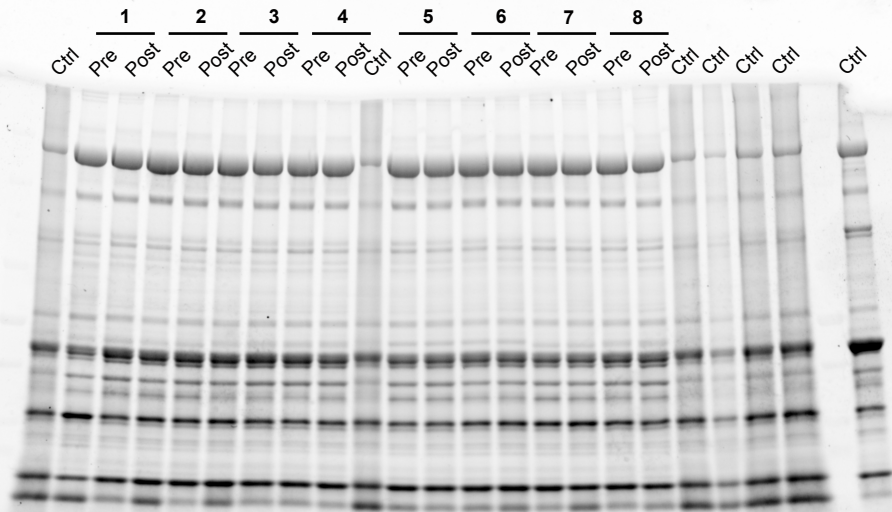

Supplement: Figure 7—figure supplement 1—source data 1. [file elife-69802-fig7-figsupp1-data1.pdf]
