# Supplementary material for: High-intensity interval training remodels the proteome and acetylome of human skeletal muscle
Source: eLife. 2022 May 31;11:e69802. doi: 10.7554/eLife.69802 (PMC9154743; doi:10.7554/eLife.69802)
Supplement: Figure 5—source data 13. [file elife-69802-fig5-data13.pdf]

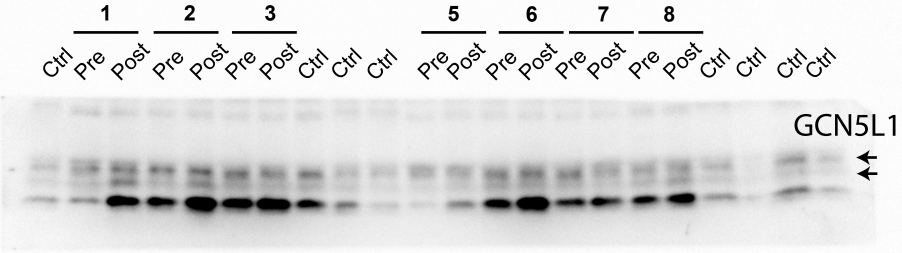
1
2
3
5
6
7
8
Ctrl
Pre
Post
Pre
Post
Pre
Post
Ctrl
Ctrl
Ctrl
Pre
Post
Pre
Post
Pre
Post
Pre
Post
Ctrl
Ctrl
Ctrl
Ctrl
GCN5L1
